# Validation of three-dimensional diffraction contrast tomography reconstructions by means of electron backscatter diffraction characterization

**DOI:** 10.1107/S002188981301580X

**Published:** 2013-07-18

**Authors:** Melanie Syha, Andreas Trenkle, Barbara Lödermann, Andreas Graff, Wolfgang Ludwig, Daniel Weygand, Peter Gumbsch

**Affiliations:** a Institute of Applied Materials, Karlsruhe Institute of Technology, Kaiserstrasse 12, 76128 Karlsruhe, Germany; b Fraunhofer Institute for Mechanics of Materials, Walter-Hülse-Strasse 1, 06120 Halle, Germany; c European Synchrotron Radiation Facility, 6 Rue Jules Horowitz, 38000 Grenoble, France

**Keywords:** microstructure characterization, diffraction contrast tomography, electron backscatter diffraction

## Abstract

Microstructure reconstructions resulting from diffraction contrast tomography data of polycrystalline bulk strontium titanate were reinvestigated by means of electron backscatter diffraction (EBSD) characterization. Corresponding two-dimensional grain maps from the two characterization methods were aligned and compared, focusing on the spatial resolution at the internal interfaces. The compared grain boundary networks show a remarkably good agreement both morphologically and in crystallographic orientation. Deviations are critically assessed and discussed in the context of diffraction data reconstruction and EBSD data collection techniques.

## Abstract 2

Corresponding two-dimensional grain maps from the diffraction contrast tomography and electron backscatter diffraction characterization methods were aligned and compared, focusing on the spatial resolution at the internal interfaces. The compared grain boundary networks show a remarkably good agreement both morphologically and in crystallographic orientation.

## 1. Introduction

The possibility of three-dimensional microstructure characterization has been a long sought wish for materials scientists. Characterization techniques were restricted to conventional two-dimensional metallography, *i.e.* optical microscopy, electron microscopy and X-ray microscopy, for decades, and it was not until the early 1990s (DeHoff, 1983 ▶) when serial sectioning allowed the first steps towards truly three-dimensional microstructure characterization. Nowadays roboted serial sectioning (Spowart *et al.*, 2003 ▶), focused ion beam milling in dual beam instruments (Sakamoto *et al.*, 1998 ▶; Groeber *et al.*, 2006 ▶) and femtosecond-laser-based ablation techniques (Echlin *et al.*, 2012 ▶) allow for destructive access to full three-dimensional crystallographic information. At the beginning of the current century the first nondestructive three-dimensional X-ray characterization techniques were proposed. Three-dimensional X-ray diffraction microscopy (Lauridsen *et al.*, 2001 ▶; Poulsen, 2004 ▶; Suter *et al.*, 2006 ▶) and X-ray diffraction contrast tomography (DCT) (Johnson *et al.*, 2008 ▶; Ludwig *et al.*, 2009 ▶) as well as differential aperture X-ray microscopy (Larson *et al.*, 2002 ▶) allow for nondestructive acquisition and reconstruction of three-dimensional grain microstructures. DCT uses a fast, truly three-dimensional acquisition procedure and shares a common experimental setup with X-ray microtomography, which allows straightforward combination with X-ray phase contrast tomography (PCT) (Cloetens *et al.*, 1997 ▶). However the DCT grain reconstruction procedure, based on two-dimensional X-ray projection topographs, is founded on a number of simplifying assumptions (kinematical diffraction, absence of orientation and elastic strain gradients inside grains) (Ludwig *et al.*, 2009 ▶) and therefore requires thorough validation by means of comparison against standard two-dimensional grain mapping techniques. After a first successful application of DCT to ceramic microstructures on the model system of strontium titanate (Syha *et al.*, 2011 ▶), the quality of the reconstructed microstructure will be critically assessed here. To this end, cross sections of reconstructed DCT microstructures are compared with grain maps provided by EBSD, a technique providing a substantially better spatial resolution down to some tens of nanometres (Humphreys, 2004 ▶). Such higher resolution is especially important at the grain boundaries. Here, the reconstructed DCT microstructures are somewhat uncertain because of a morphological dilation step in the reconstruction process.

## 2. Experimental

### 2.1. Sample preparation

Strontium titanate specimens were prepared from 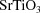 powders processed by the mixed oxide route from 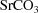 and 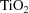 (both 99.9+%, Sigma Aldrich Chemie, Taufkirchen, Germany) using a molar Sr/Ti ratio of 0.996. After several milling and calcining steps, a cylindrical shape and final green density were obtained by uniaxial and isostatic pressing procedures. Sintering for 1 h at 1873 K in oxygen atmosphere yielded a material with an average grain radius of 14.1 (15) µm; the specimen was then cut and ground to a columnar shape of approximately 300 µm in diameter and 380 µm in height. Following a first DCT investigation, the specimen was annealed for another hour with identical sintering conditions. Detailed information on the fabrication and annealing of the specimens and of microstructure investigations are provided elsewhere (Bäurer, Weygand *et al.*, 2009 ▶; Bäurer, Kungl & Hoffmann, 2009 ▶; Syha *et al.*, 2011 ▶).

### 2.2. Diffraction and phase contrast tomography

The specimen was subjected to X-ray diffraction contrast tomography measurements using the setup and technical details described elsewhere (Johnson *et al.*, 2008 ▶; Ludwig *et al.*, 2009 ▶; Reischig *et al.*, 2013 ▶). The X-ray DCT experiments were performed at the Materials Science Beamline ID11 of the European Synchrotron Radiation Facility (ESRF). Data acquisition was performed during full 360° scans with an angular stepping of 0.05°, taking in total 7200 images. This rendered the exploitation of Friedel pairs during microstructure reconstruction possible. In the course of the data analysis procedure, diffraction spots are segmented from the raw images and pairs of diffraction spots that are 180° separated are identified (Ludwig *et al.*, 2009 ▶; Johnson *et al.*, 2008 ▶; Reischig *et al.*, 2013 ▶). Groups of these Friedel pairs belonging to the same grain are then collected and individual grains are reconstructed using an algebraic tomographic reconstruction algorithm  (Batenburg *et al.*, 2010 ▶; Batenburg & Sijbers, 2011 ▶). After assembly of the grain volumes into the common sample volume, the resulting grain map will contain small regions close to grain boundaries and triple junctions which remain unassigned (Ludwig *et al.*, 2009 ▶). In order to ensure the assembly of these reconstructed individual grains to fill the entire volume, a uniform dilation procedure based on the dilate function of the MATLAB (The MathWorks Inc., Natick, MA, USA) image processing toolbox is applied. In the voxelated reconstruction of the microstructure, voxels belonging to a given grain have a unique gray value (label), and for each of the grains the crystallographic orientation is stored in an accompanying data structure. In parallel to the collection of diffraction signals used for grain reconstruction, conventional absorption images are used to reconstruct the attenuation coefficient in the sample volume. Attenuation contrast does not allow for detection of porosities below a certain size – in our case about 3–5 µm. Therefore, the information was complemented by PCT (Cloetens *et al.*, 1997 ▶). A separate PCT data set was acquired using the same experimental setup but a larger sample–detector distance. The free-space propagation leads to edge enhancement (Fresnel diffraction) which increases the visibility of small pores. The resulting three-dimensional reconstruction of porosities inside the specimen is superimposed with the DCT reconstruction, so that the pores are recovered after the dilation step of the DCT reconstruction which has generated a dense volume. With a voxel size of 0.7 µm, the spatial resolution of the final reconstruction is of the order of 1 µm.

### 2.3. EBSD characterization

**Figure 1:**
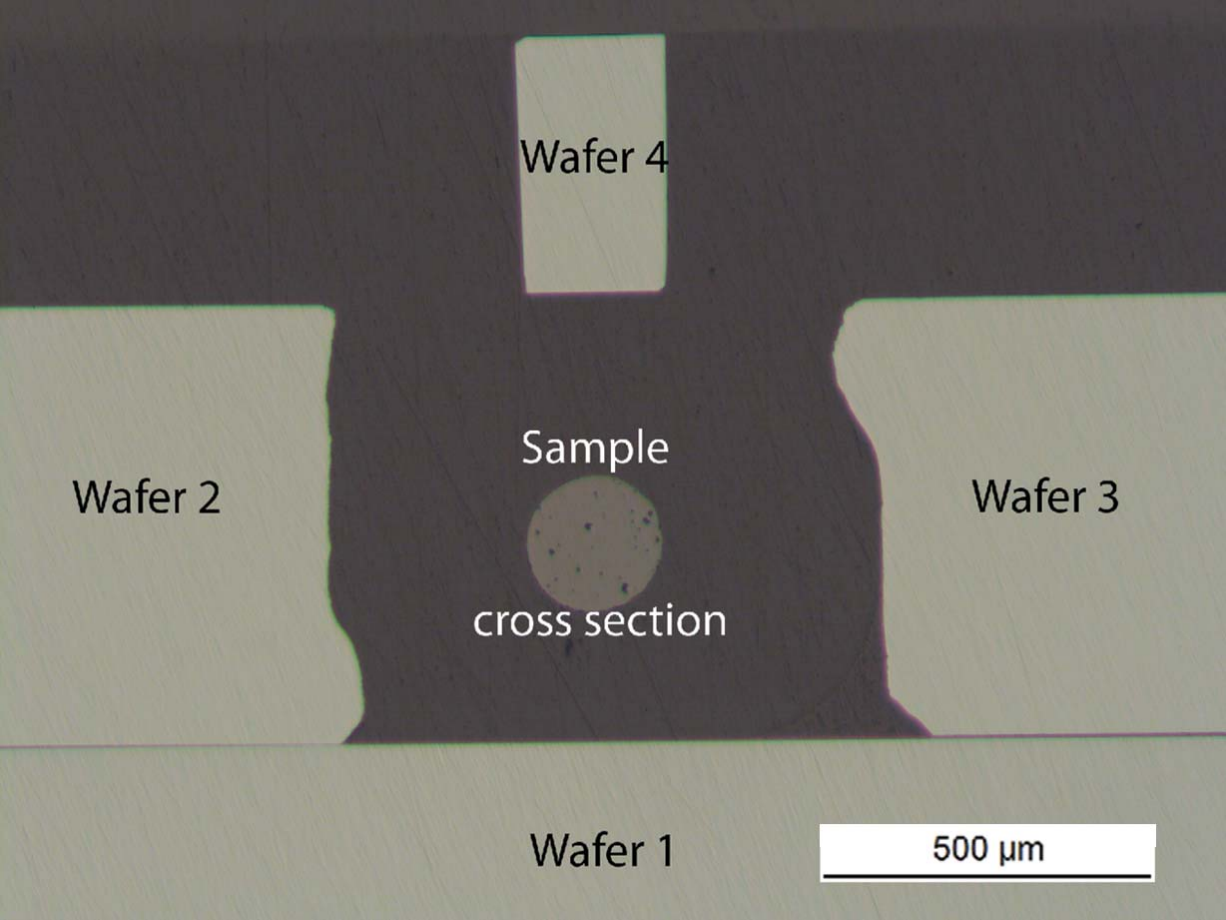
Optical micrograph of sample and silicon embedded in epoxy resin.

After DCT measurements the 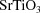 specimen was sectioned for EBSD analysis approximately perpendicular to the cylinder axis. As illustrated in Fig. 1 ▶, the 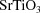 specimen was fixed onto a silicon wafer by two additional wafer pieces. A fourth wafer was mounted with its surface parallel to the first wafer with a 90° edge parallel to the sample normal. This assembly was embedded in epoxy resin.

The four wafer pieces improve the electrical conductivity and the mechanical stability and help to align the sample before each EBSD measurement. The edge-on fourth wafer was used for depth measurement during the mechanical cross sectioning. The sectioning was performed by mechanical grinding and polishing with diamond suspensions of 3 and 1 µm grain size followed by an oxide polishing suspension. Finally, the sections were ion polished at 5 kV and an incident angle of 8° and covered with a thin carbon film to improve electrical conductivity. A total of eight sections were prepared by eroding layers of 3–10 µm thickness in each grinding step. The sections stay nearly plane parallel during the preparation owing to the comparatively large diameter of the embedding. The EBSD measurements were realized in a scanning electron microscope (Zeiss; Supra 55 VP) at 15 kV equipped with an EBSD system (EDAX TSL). The sample was mounted with 70° tilt to the EBSD detector on an inclined sample holder at a working distance of 11 mm. The measurements were made on the entire cross section in a hexagonal grid with 1 µm step size. Diffraction patterns were acquired with an 8 × 8 binning at a camera read out time of 0.01 s. This leads to a rate of mapping of about 70 patterns per second. The actual lateral resolution of the EBSD technique is higher than the chosen step size. It depends on the energy of the primary electrons, the defect density of the material and the orientation of the sample (Isabell & Dravid, 1997 ▶; Zaefferer, 2007 ▶). The cubic perov­skite structure with a lattice constant of 0.3905 nm and space group 221 was used for indexing the diffraction patterns. Grains were identified if they had a size of at least three pixels and a misorientation inside the grain of less than 3°. Grain boundary networks were generated using the *OIM* software package (EDAX Inc., Mahwah, NJ, USA). Post-processing involved scaling of the total image and unidirectional stretching to correct for tilt. Prior to pore detection, which was done on color images of the orientation maps, image noise was reduced using the 5 × 5 median filter implemented in MATLAB.

### 2.4. Identifying corresponding cross sections

**Figure 2:**
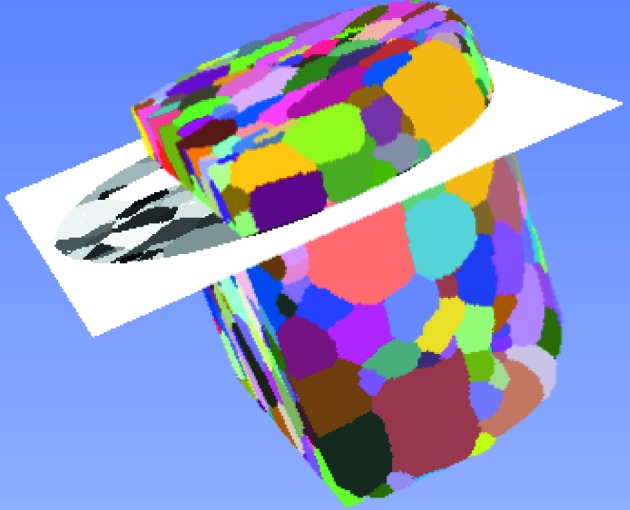
Three-dimensional volume of the sample and identified cutting plane.

Cross sections through the reconstructed DCT structure that match the EBSD sections were identified from the spatial distribution of the pores. As a result of the serial sectioning process, the slices through the specimen are slightly tilted with respect to the cylinder axis, and the resulting images originate from unknown cutting planes. It is therefore necessary to identify the orientation of the EBSD images with respect to the voxelized DCT data and to create an artificial cut through the reconstructed volume to allow for comparative validation. Beyond the misorientation, the cross sections could also be slightly bulged as a result of the mechanical polishing. However this bulging mostly occurred at the edges of the sample and was therefore not specifically corrected for. First, the spatial distribution of the pores was used to obtain a preliminary estimation of the cross section. To determine a more precise orientation, preferably small round-shaped pores that lie enclosed in grains and extend only over few (*e.g.* 1–3) transverse slices in the DCT reconstruction were considered. Their nearly spherical shape and small spatial extent makes the approximate center of these pores suitable input data for a plane fitting approach. Starting from the EBSD cross section data, corresponding pores were identified manually in the reconstructed structure using distinct patterns of neighboring pores, and the plane orientation was fitted by means of a least-squares approach. The obtained plane orientation was used to visualize the final slices. Fig. 2 ▶ shows the three-dimensional volume and one of the identified cutting planes.

## 3. Results

**Figure 3:**
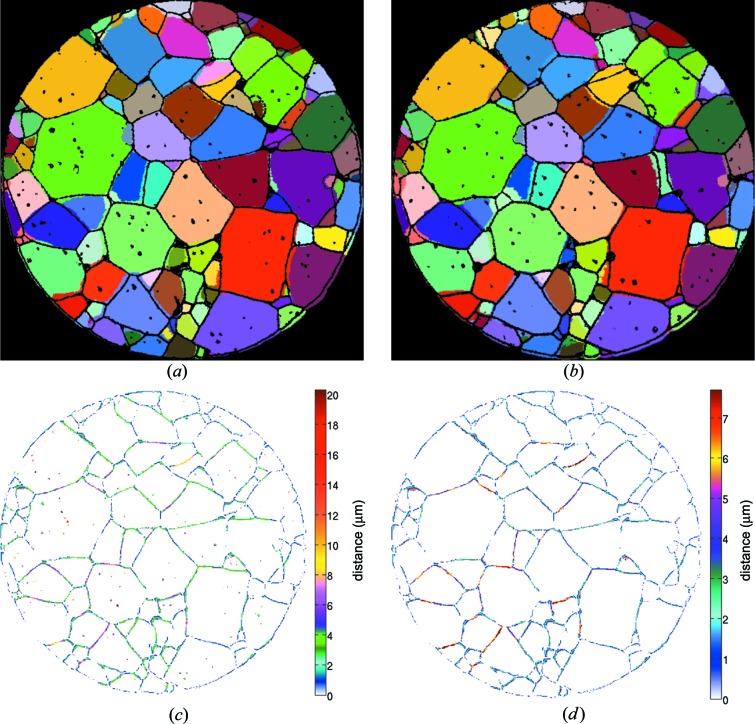
Two different cross sections at heights of 308 µm (*a*) and 311 µm (*b*) in the DCT reconstruction (colored) superimposed with the corresponding EBSD networks. (*c*), (*d*) The DCT network shown in (*a*) colored according to Euclidean distance to the corresponding EBSD network, respectively, with and without pores.

Figs. 3 ▶(*a*) and 3 ▶(*b*) show superpositions of EBSD characterizations of two cross sections and the closest matching sections of the reconstructed tomography data. Here, the EBSD data are pictured as a grain boundary network, while the colored grains show the DCT reconstruction. The spacing between the two EBSD sections is approximately 3 µm.

The compared grain boundary networks in Fig. 3 ▶(*a*) contain 107 (DCT) and 108 grains (EBSD). The average grain size for these sections was measured by the linear intersect method and found to be 30.1 (4) µm (DCT) and 29.7  (4) µm (EBSD). The compared grain boundary networks in Fig. 3 ▶(*b*) show cross sections containing 109 (DCT) and 110 grains (EBSD). The average grain size for these sections was found to be 31.5 (6) µm (DCT) and 28.9 (5) µm (EBSD). A good agreement in overall grain shape and pore size was found for both sections presented in this work.

**Figure 4:**
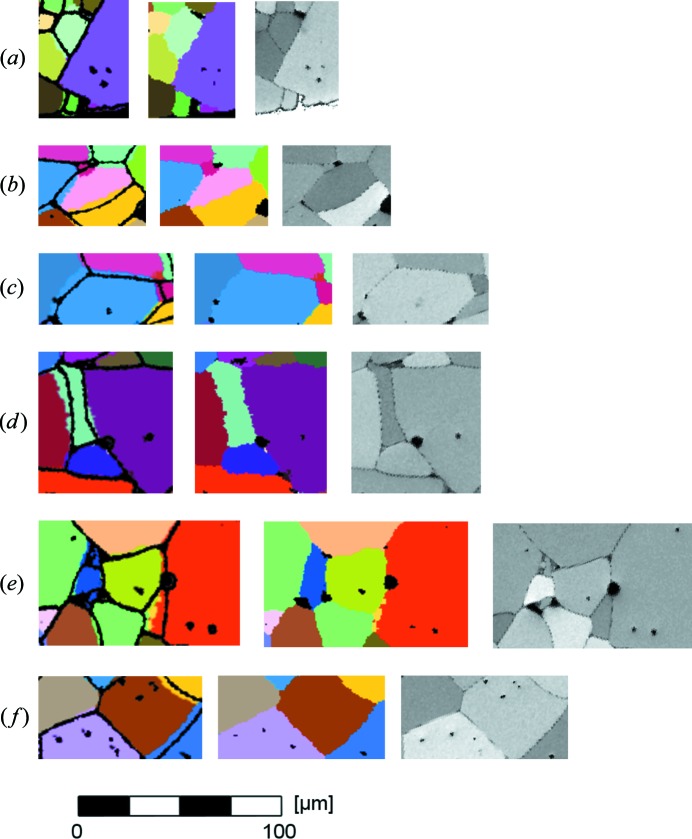
(*a*)–(*f*) Close-ups of several regions selected from Figs. 3 ▶(*a*) and 3 ▶(*b*). DCT cross section superposed with the grain boundary network obtained from the EBSD image (left), cross section through the DCT reconstruction (center) and monochrome version of the EBSD orientation map (right).

Visual comparison of corresponding slices reveals a higher number of pores in the EBSD data compared with the equivalent cross section in the combined grain map (*i.e.* the map resulting from merging the initial DCT grain map with the porosity map determined from PCT; for simplicity, we will refer to this combined map as the ‘DCT map’ in the rest of the manuscript). The number of pores in the DCT map reaches approximately 80–90% of the pore count in the corresponding EBSD section. Notably, even small pores (*i.e.* covering an area smaller than 10 µm^2^ in the EBSD data) could also be detected in the DCT data. Upon visual inspection, the overall shape of the grains is in excellent agreement. The average error between the corresponding grain boundary networks was estimated using Euclidean distance mapping (Danielsson, 1980 ▶) and found to be 1.98 µm for the cross section shown in Fig. 3 ▶(*a*) and 1.95 µm for the cross section shown in Fig. 3 ▶(*b*). After removal of unidentified intragranular pores, these values changed to 1.86 and 1.88 µm, respectively. The pore-adjusted DCT network of the cross section presented in Fig. 3 ▶(*a*) is reprinted and colored according to the Euclidean distance to the EBSD network in Figs. 3 ▶(*c*) and 3 ▶(*d*) with and without pores, respectively. The distance image reveals that the grain boundaries in the DCT reconstruction appear to be slightly more curved, since the greatest deviation between DCT and EBSD networks is typically located on the grain boundaries rather than at the triple points. Fig. 4 ▶ shows close-ups of regions chosen from both inspected cross sections. The superposed representation of DCT grain map and EBSD grain boundary network is complemented by both the DCT (colored) and the EBSD (monochrome) grain maps to allow for a more detailed investigation. From these images it is clearly visible that the detection of small pores located inside grains by DCT is not completely reliable. While we found corresponding intragranular pores in many cases (Figs. 4 ▶ *a*, 4 ▶ *d*, 4 ▶ *e* and 4 ▶ *f*), there are also a few examples of pores revealed in the EBSD analysis that are not resolved in the DCT reconstruction [*e.g.* missing pores in the brown grain in Fig. 4 ▶(*b*), the blue grain in Fig. 4 ▶(*c*), the upper left green grain in Fig. 4 ▶(*e*) or the brown grain in Fig. 4 ▶(*f*)]. Some pores appear to be larger in the superposed image than in the monochrome version of the EBSD orientation map. This is both a linewidth effect and a filtering artifact arising when extracting pores from the orientation maps. Moreover, we see a pixel-wise smearing at the interfaces in some of the DCT reconstructions. These artifacts occur typically as one-voxel-wide extrusions reaching two or three voxel lengths into the neighboring grain (Figs. 4 ▶ *b*, 4 ▶ *d* and 4 ▶ *e*). Fig. 4 ▶(*e*) reveals a region of very small grains in the EBSD grain map that is not resolved in DCT (blue region in the left part of the image). Furthermore, we obtain a local deviation in curvature. In these cases, the grain boundaries appear to be more straight in the EBSD grain maps than in the DCT maps [*e.g.* between the blue and pink grains in Fig. 4 ▶(*b*) or between the blue grains in Fig. 4 ▶(*c*)], while Fig. 4 ▶(*a*) shows that the surface contours of the two grain maps are nearly identical. Lastly, we obtained some pores at doubtable locations in the DCT reconstruction [*e.g.* the pore inside the pink grain in the upper part of Fig. 4 ▶(*d*)]. Both the EBSD grain map and the shape of the pore suggest it to be intergranular rather than intragranular. The identification of the smallest misorientation between the orientation of 24 crystallites obtained by both characterization methods reveals a consistent transformation between EBSD and DCT orientation space for all the crystallites, which was found to be a rotation of 20.8 (15)° around the misorientation axis.

## 4. Discussion

The goal of nondestructive three-dimensional imaging of microstructure evolution in ceramic materials can be realized *via* X-ray diffraction contrast tomography. This technique provides grain maps obtained from a complex reconstruction and post-processing procedure. We investigated the quality of these grain maps using the example of an 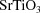 specimen. The reconstructed microstructure was validated against EBSD data of the specimen. Therefore, grain boundary networks have been extracted from EBSD data and have been compared with corresponding two-dimensional cross sections through the reconstructed volume. The number of grains found in the EBSD and corresponding DCT maps is almost identical, with a good agreement in average grain size. The slight difference in the counting of grains (we find one grain more in the EBSD analysis of both cross sections) is the result of a number of grains being barely cut in either of the cross sections, thus appearing as really small areas. The smallest detected grain has a radius of about 2.21 µm in the undilated state and 2.72 µm in the dilated state, giving a realistic estimation for the resolution limit for the reconstruction of small grains. For the purpose of noise reduction, diffraction spots with an area smaller than about 100 pixels have been excluded from the current analysis. As a consequence, grains below the corresponding volume (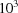 voxels) cannot be indexed and are lost in the analysis. Thus it is more likely that fewer grains will be observed in the DCT maps. In addition, a small angular uncertainty in the plane fitting might leave very small grains undetected above or below the cutting plane.

Finding a good agreement in overall grain shape, we observed an average Euclidean distance between the grain boundary networks obtained by DCT and EBSD that corresponds well to the previously reported accuracy of 1.6 µm obtained from a three-dimensional distance transform (Ludwig *et al.*, 2009 ▶). It should be emphasized that an estimate made from a two-dimensional distance transform on a three-dimensional grain boundary network is necessarily conservative. For grain boundaries that are close to parallel to the two-dimensional cross section, a one-pixel error in the three-dimensional reconstruction may give rise to large shifts and irregular shapes in the corresponding two-dimensional observation. The brown grain at the bottom of Figs. 3 ▶(*a*) and 3 ▶(*b*) and the light-green grain at the center of Fig. 4 ▶(*d*) are examples of this kind of configuration. The remaining differences in grain boundary location, which tend to appear half way between two triple points, can partly be explained by the dilation procedure applied during post processing of the diffraction data. This dilation, being of uniform character, is likely to hinder the occurrence of the pronounced faceting that could be expected in anisotropic materials, as can be seen in the purple grain in Fig. 4 ▶(*d*). Replacing the up-to-date uniform dilation step with a more elaborate algorithm based on forward modeling of the diffraction process could represent a starting point for improved microstructure reconstructions with respect to the spatial resolution at the grain boundaries. For each of the unassigned voxels, the goodness of fit of simulated *versus* measured diffraction patterns can be evaluated while assigning any of the possible (adjacent) grain orientations. Another fact that might account for some of the deviations in the grain boundary networks is a distortion of unknown size resulting from the EBSD measurements. The comparison of Euclidean distance transforms with and without pores shows clearly that, despite the higher resolution resulting from PCT data sets, the porosity data of the two characterization methods are not yet reliably comparable. This phenomenon originates from several factors: The higher number of (especially intragranular) pores in the EBSD slices can be related to ring artifacts and uncertainties in the PCT reconstructions  (Cloetens *et al.*, 1997 ▶). The artifacts hamper the segmentation of individual pores used for the final assembly of the reconstructed sample volume. Additionally a threshold was applied that eliminated small pores and pores with a low intensity value. Furthermore, thin material layers above pores might have collapsed during the mechanical preparation of the 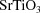 specimen for sectioning, thereby revealing underlying pores, so that the EBSD sections might also reveal pores that are underneath the actual section. Moreover, material that has been removed during polishing might accumulate in large pores, altering the shape of the pores visible in the EBSD orientation map. Obviously, the manual preparation of the specimen cannot guarantee a perfectly even cutting plane and thereby accounts for some inaccuracy in the comparison of corresponding cross sections. This comes into effect especially in the peripheral areas of the EBSD sections. The interpolation procedure applied during plane cutting might account for the small artifacts that have been obtained at the grain boundaries in the DCT grain map. Considering the shape of the reconstructed porosities, it is noteworthy that spherical small pores seem to lie preferably within grains, whilst larger eccentrically shaped pores seem to appear at triple lines. This information could be exploited in an improved version of the dilation algorithm, for example, in creating a probability map for grain and pore areas. High-resolution porosity information could consolidate the accuracy of this approach. Better resolved three-dimensional images (0.3 µm pixel size ≃ 1 µm full width at half-maximum of detector point spread function) can be obtained using a different combination of (microscope) optics in the high-resolution detector system employed for (parallel beam) PCT measurements. X-ray microscopy techniques (*e.g.* zoom tomography; Mokso *et al.*, 2007 ▶) can provide higher spatial resolution. A 2048^2^ zoom tomography reconstruction with a voxel size of 150 nm would still allow analysis of the 300 µm-diameter sample employed in the current study. Despite the possible further improvements in the DCT reconstruction, the currently obtained accuracy in the determination of grain shape and size of about 1.5 µm seems to be good enough to directly compare grain growth simulations and DCT observations for reasonably large grain growth regimes. The accuracy may not yet be sufficient to identify general anisotropies in grain boundary properties except for very strong cases of faceting. Owing to the high mapping rate chosen to prevent electrical charging of the sample, the orientation resolution in the current EBSD investigation (1°) is inferior to the resolution in orientation determination from DCT investigations (0.1°). Nevertheless, the existence of a unique transformation between the grain orientations in the two representations proves that EBSD measurements are a suitable tool for validating both structural and crystallographic three-dimensional DCT data.

## 5. Conclusion

The applied DCT technique is an appropriate tool to nondestructively characterize three-dimensional microstructures in ceramics with the added benefit of high-resolution grain orientations. The applied instruments and reconstruction techniques yield very good results in terms of image quality and spatial resolution and excellent orientation resolution. However, an optimization of the processing of the diffraction data and the use of elaborate optical techniques (*e.g.* zoom tomography), as mentioned in the discussion, are desirable in order to improve the spatial resolution at the grain boundaries and to improve accuracy with respect to curvature and porosity. The accuracy of the EBSD validation might be further improved if the material could be ablated *in situ* to a defined thickness using a machine grinding technique. The recently developed TriBeam technique  (Echlin *et al.*, 2012 ▶) provides plane-parallel *in situ* laser ablation which is significantly faster than the EBSD scan itself and thus allows fast acquisition of large (several hundred slices) three-dimensional post-mortem data sets. Validation against such data is necessary and will help to improve the reconstruction technique further. Finally, we can say that the concept of DCT measurements is applicable to perovskite ceramics, resulting in three-dimensional grain structures with reasonable resolution at the grain boundaries and high-accuracy orientation detection. Future tasks should focus on improvement of the spatial resolution of grain boundaries and the application of DCT to acquire time-resolved microstructure characterizations (*e.g.* annealing or sintering experiments).

## References

[bb1] Batenburg, K. J. & Sijbers, J. (2011). *IEEE Trans. Image Process.* **20**, 2542–2553. 10.1109/TIP.2011.2131661 21435983

[bb2] Batenburg, K. J., Sijbers, J., Poulsen, H. F. & Knudsen, E. (2010). *J. Appl. Cryst.* **43**, 1464–1473.

[bb3] Bäurer, M., Kungl, H. & Hoffmann, M. (2009). *J. Am. Ceram. Soc.* **92**, 601–606.

[bb4] Bäurer, M., Weygand, D., Gumbsch, P. & Hoffmann, M. (2009). *Scr. Mater.* **61**, 584–587.

[bb5] Cloetens, P., Pateyron-Salomé, M., Buffière, J. Y., Peix, G., Baruchel, J., Peyrin, F. & Schlenker, M. (1997). *J. Appl. Phys.* **81**, 5878–5887.

[bb6] Danielsson, P. (1980). *Comput. Graphics Image Process.* **14**, 227–248.

[bb7] DeHoff, R. (1983). *J. Microsc.* **131**, 259–263.

[bb8] Echlin, M. P., Mottura, A., Torbet, C. J. & Pollock, T. M. (2012). *Rev. Sci. Instrum.* **83**, 023701. 10.1063/1.3680111 22380093

[bb9] Groeber, M., Haley, B., Uchic, M., Dimiduk, D. & Ghosh, S. (2006). *Mater. Charact.* **57**, 259–273.

[bb10] Humphreys, F. (2004). *Scr. Mater.* **51**, 771–776.

[bb11] Isabell, T. & Dravid, V. (1997). *Ultramicroscopy*, **67**, 59–68.

[bb12] Johnson, G., King, A., Honnicke, M. G., Marrow, J. & Ludwig, W. (2008). *J. Appl. Cryst.* **41**, 310–318.

[bb13] Larson, B. C., Yang, W., Ice, G. E., Budai, J. D. & Tischler, J. Z. (2002). *Nature*, **415**, 887–890. 10.1038/415887a 11859363

[bb14] Lauridsen, E., Schmidt, S., Margulies, L., Poulsen, H. & Jensen, D. J. (2001). *Recrystallization and Grain Growth. Proceedings of the First Joint International Conference August 27–31, 2001*, edited by G. Gottstein & D. A. Molodov, pp. 589–594. Berlin, Heidelberg: Springer-Verlag.

[bb15] Ludwig, W., Reischig, P., King, A., Herbig, M., Lauridsen, E. M., Johnson, G., Marrow, T. J. & Buffière, J. Y. (2009). *Rev. Sci. Instrum.* **80**, 033905. 10.1063/1.3100200 19334932

[bb16] Mokso, R., Cloetens, P., Maire, E., Ludwig, W. & Buffière, J.-Y. (2007). *Appl. Phys. Lett.* **90**, 144104.

[bb17] Poulsen, H. (2004). *Three-dimensional X-ray Diffraction Microscopy. Mapping Polycrystals and Their Dynamics.* Berlin, Heidelberg: Springer.

[bb18] Reischig, P., King, A., Nervo, L., Viganó, N., Guilhem, Y., Palenstijn, W. J., Batenburg, K. J., Preuss, M. & Ludwig, W. (2013). *J. Appl. Cryst.* **46**, 297–311.

[bb19] Sakamoto, T., Cheng, Z., Takahasi, M., Owari, M. & Nihei, Y. (1998). *Jpn. J. Appl. Phys.* **37**, 2051–2056.

[bb20] Spowart, J., Mullens, H. & Puchala, B. (2003). *J. Miner. Met. Mater. Soc.* **55**, 35–37.

[bb21] Suter, R., Hennessy, D., Xiao, C. & Lienert, U. (2006). *Rev. Sci. Instrum.* **77**, 123905.

[bb22] Syha, M., Rheinheimer, W., Bäurer, M., Lauridsen, E., Ludwig, W., Weygand, D. & Gumbsch, P. (2011). *Scr. Mater.* **66**, 1–4.

[bb23] Zaefferer, S. (2007). *Ultramicroscopy*, **107**, 254–266. 10.1016/j.ultramic.2006.08.007 17055170

